# G1359A Polymorphism of the Cannabinoid Receptor 1 Is Not Associated with Overweight and Dyslipidemia in Young Northeastern Mexicans

**DOI:** 10.7759/cureus.5776

**Published:** 2019-09-26

**Authors:** Ismael Rodríguez-Rodríguez, Karla Fernández-Quiroga, Pedro Araujo-Moreno, Isaías Balderas-Rentería, Omar Gonzalez-Santiago

**Affiliations:** 1 Chemical Science, Jagiellonian University, Kraków, POL; 2 Chemical Science, Universidad Autónoma De Nuevo León, San Nicolas de los Garza, MEX; 3 Chemical Science, Universidad Autónoma De Nuevo León, Monterrey, MEX; 4 Chemical Science, Universidad Autonoma De Nuevo Leon, San Nicolas de los Garza, MEX

**Keywords:** gene polymorphism, cannabis receptor, serum lipids, mexico, young

## Abstract

There is extensive evidence to believe that the endocannabinoid system plays an important role in energy homeostasis through a variety of mechanisms. This study aimed to analyze the association between polymorphism rs12720071 of the cannabinoid type 1 receptor (CNR1) gene with dyslipidemia and overweight in young, healthy Mexicans. The association was analyzed with a logistic regression model and expressed as odds ratio (OR). A total of 148 individuals agreed to participate. Overall, the serum concentrations of lipids were found to be in the normal range. However, females presented higher levels of cholesterol and low-density lipoprotein (LDL) than males [probability value (p) = <0.05]. In addition, females presented higher risk of being overweight (BMI: >25) [OR = 3.57; 95% confidence interval (CI): 1.05-12.20; p = 0.04], than males. Our results suggest that this polymorphism could influence BMI in young females.

## Introduction

Mexican diet has been changing constantly over the last few years [1]. This shift in diet habits, along with a sedentary culture and increased food intake, has caused Mexicans to develop conditions such as metabolic syndrome, obesity, and overweight, which are risk factors for a variety of chronic diseases like diabetes mellitus, dyslipidemia, and cardiovascular diseases [2,3].

Overweight and obesity have become an important public health problem in Mexico, and they affect both genders and all age groups [4]. It is estimated that by 2050, the percentage of people of normal weight in México will be only 12 % and 9 % of the population for males and females, respectively. It is projected that the obesity and the overweight epidemic would burden the country by $US 1.7 billion in healthcare-related costs by 2050 [5]. The number of individuals who are obese and overweight worldwide is on the rise despite massive efforts to control this hazardous epidemic [6].

The existing evidence suggests that the endocannabinoid system (ECS) is a critical factor in obesity and metabolism, and has been proposed as an appropriate molecular target for efficient anti-obesity treatment [7,8]. The ECS is comprised of the G-coupled cannabinoid receptor types 1 and 2 (CB1 and CB2), the endogen endocannabinoids anandamide (AEA) and 2-arachidonoyl glycerol (2-AG), and the responsible enzymes for their synthesis and degradation [9]. At the central level, cannabis receptors activate both homeostatic and hedonic food-intake pathways in diverse areas of the central nervous system [10]. It also induces the release of various orexigenic and anorexigenic neuropeptides that regulate food-seeking behavior [11,12].

The cannabinoid type 1 receptor (CNR1) gene that encodes the CB1 receptor is located at 6q14-q15 loci, and it contains 4 exons and 5 introns. The region that encodes the CB1 receptor is located at the 5’ end of the exon 4, and the complete gene is 26.1 kb [13]. Reported genetic variants of this gene have proved to be of great relevance due to their association with anthropometric measures of obesity, metabolic disorders, and dyslipidemia, as recently reported in the European [14,15], Caucasian [16,17], Asian [18,19], and southern Brazilian [20] populations.

The main objective of this study was to evaluate the association of rs12720071 polymorphism with serum concentrations of lipids [cholesterol, high-density lipoprotein (HDL), low-density lipoprotein (LDL), triglycerides, and very-low-density lipoprotein (VLDL)] and BMI in a sample of young individuals of the northeast of Mexico.

## Materials and methods

Subjects

Several young individuals of northeast Mexico were invited to participate. The event took place at the Faculty of Chemical Sciences of Universidad Autonoma de Nuevo León (UANL), Nuevo León, Mexico from January 2017 through September 2017. Each subject provided a blood sample of 5 ml after 12 hours of nocturnal fasting. The samples were stored at 4° C until their use. Each of the collected blood samples was used for lipid quantification. The inclusion criteria for this study were as follows: (1) subjects should be males or females between the age of 18 and 35; (2) subjects should sign an informed-consent form; (3) subjects should have no prior infectious or any other known diseases. The study had been approved by the institutional board review of the Faculty of Chemical Science (approval number: 04-099604-FAR-11/257).

Measurements

A physical examination was performed in each of the participants. Height (cm) and Weight (kg) were measured and registered. BMI was calculated and serum levels of total cholesterol (TC), HDL, LDL, and VLDL were quantified by spectrophotometry using biochemistry analyzer A25 (BioSystems, Barcelona, Spain).

Genotyping

Genomic DNA was isolated from blood using the phenol-chloroform method, while for genotyping, we used the polymerase chain reaction-restriction fragment length polymorphism (PCR-RFLP) method. Conditions for both methods had been previously reported by our research team [21].

Statistical analysis

Allelic and genotypic frequencies were determined by direct counting. Hardy-Weinberg equilibrium (HWE) was tested with the χ2 goodness-of-fit test. Comparisons between continuous variables groups were analyzed by Student’s t-test. The association between the genetic variants of dyslipidemia and overweight (BMI: >25) were assessed through logistic regression analyses and expressed as odds ratio (OR) with 95% confidence interval (CI). A probability value (p) of <0.05 was used to assess significance. Statistical analyses were conducted using Minitab version 18 (Minitab, LLC, State College, Pennsylvania).

## Results

From the original sample of 200 subjects, 148 agreed to participate; 78 were female and 70 were male. Lipid data of one male individual were not included in the final analysis as he did not meet the fasting requirement adequately. Although the levels of lipids were in the normal range in both genders, females showed higher levels of cholesterol (p = 0.02) and LDL (p = 0.01) than males (Table 1).

**Table 1:** Lipid profile in the sample of young Mexicans individuals HDL: high-density lipoprotein; LDL: low-density lipoprotein; VLDL: very-low-density lipoprotein *p = <0.05

	Total	Male	Female	Probability value
Age (years)	19.4	19.3	19.4	0.83
Weight (kg)	67.9	69.1	66.9	0.44
BMI (kg/m^2^)	24.3	24.6	24	0.53
Cholesterol (mg/dL)	159	152.8	164.5	0.02*
HDL (mg/dL)	57.7	57.2	58.1	0.74
LDL (mg/dL)	86.1	80	91.7	0.01*
Triglycerides (mg/dL)	80.3	82	78.8	0.61
VLDL (mg/dL)	15.9	16.1	15.8	0.76
Cholesterol/HDL	2.9	2.8	3	0.39

The sample was studied in Hardy-Weinberg equilibrium (p = 0.91) with a frequency distribution for various genotypes as follows: AA = 0.85, AG = 0.14, GG = 0.01. Because the GG genotype was only present in one subject, analyses were carried out according to the G dominant model (AA vs AG+GG). In the overall sample, there was no significant difference between AA and AG+GG in lipid profile or BMI. Female carriers of G allele showed higher BMI and lower levels of HDL than those of AA genotype; however, the HDL levels were within the normal range in both genders (Table 2).

**Table 2:** Anthropometric variables and serum lipid levels according to genotypes HDL: high-density lipoprotein; LDL: low-density lipoprotein; VLDL: very-low-density lipoprotein; AA, AG, AG: genotypes *p = <0.05

	Total			Male			Female		
Variable	AA (N=125)	AG+GG (N=22)	Probability value	AA (N=61)	AG+GG (N=9)	Probability value	AA (N=64)	AG+GG (N=13)	Probability value
Age (years)	19.4	19.1	0.37	19.3	19.3	0.98	19.5	19	0.28
Weight (kg)	67.1	73	0.16	69	69.9	0.9	65.2	75.1	0.06
BMI (kg/m^2^)	24	26.2	0.09	24.7	24	0.75	23.3	27.7	<0.01*
Cholesterol (mg/dL)	160.4	150.7	0.17	153.8	146.6	0.47	166.8	153.5	0.19
HDL (mg/dL)	58.5	53.2	0.15	57.1	57.9	0.9	59.8	49.9	0.03*
LDL (mg/dL)	86.6	83.7	0.67	80.5	76.3	0.65	92.3	88.9	0.72
Triglycerides (mg/dL)	82.3	69	0.12	84.9	62	0.13	79.8	73.8	0.54
VLDL (mg/dL)	16.3	13.8	0.14	16.7	12.3	0.15	16	14.8	0.55
Cholesterol/HDL	2.9	2.9	0.94	2.9	2.5	0.32	2.9	3.1	0.45

According to the results of the logistic regression analyses, the rs12720071 polymorphism is not associated with dyslipidemia [OR = 0.22 (95% CI: 0.02-1.74)] nor overweight [OR = 1.49 (95% CI: 0.59-3.77)]. However, a significant association was found between this polymorphism and a higher risk of being overweight in women carrying the G allele [OR = 3.57 (95% CI: 1.04-12.19)] (BMI = 25 kg / m2 as the cutoff point).

## Discussion

Obesity is a major health issue caused by genetic and environmental factors [22]. Since some studies have associated the ECS with different obesity-related conditions, it has become a potential target for new genotype-phenotype association studies, offering a new perspective on how genetics plays an important role in people acquiring this condition [23].

In this study, we quantified serum lipid levels in a sample of young Mexicans who had previously been genotyped in the CNR1 gene. We believe this is the first study about the association between cannabis receptor polymorphism and lipid levels in a sample of young individuals (18-25 years old). The calculated allelic frequency of the genotype was consistent with those reported for a Mexican-ancestry population from Los Angeles, California [24], as well as with those reported in a Caucasian population [25].

Overall, there was no significant difference between AA and AG+GG genotypes of rs12720071 polymorphism in the lipid profile and BMI. This finding suggests that this polymorphism probably has no impact on the lipid metabolism of young Mexican individuals. However, previous studies in adults of other countries indicate that carriers of the G allele present obesity traits such as a higher android fat deposit [14], increased subscapular skinfold thickness and waist circumference [26]. In fact, the literature regarding this genetic variant appears to be inconsistent since other studies have reported no association between it and any adiposity-related trait [20,27], such as nonalcoholic fatty liver disease in women with polycystic ovary syndrome [28]. In addition, a study has shown a lower prevalence of the metabolic syndrome in those subjects carrying the G allele (OR: 0.398; p = 0.003), pointing it out as an independent predictive factor for the lack of this syndrome [27]. These inconsistencies could be explained by a wide number of environmental factors such as lifestyle, diet, gender, age, and genetic background.

An interesting finding of this study was the significant difference between AA and AG+GG genotypes of females. Carriers of allele G showed higher BMI and lower levels of HDL than the AA genotype. This finding suggests that, unlike men, the polymorphism studied may affect BMI and lipid profile of women. However, more studies are needed since other factors such as sex hormones may have greater effect than polymorphism studied. In this sense, it should be remembered that the adverse effects of cannabis consumption are more pronounced in women than men [29].

A limitation of our study was the small sample size, which could have lead to low statistical power and may explain the lack of any findings related to association with dyslipidemia and overweight in man. Another limitation was the number of analyzed genetic variants in our study. We assessed only one polymorphism of the CNR1 gene, but there are many others that have been recently implicated with adverse metabolic profiles and obesity characteristics.

## Conclusions

We found that the G1359A polymorphism of the CNR1 gene is not associated with overweight and dyslipidemia in the young Mexicans we studied. However, female carriers of G allele present higher BMI and lower levels of HDL than those of AA genotype. The ongoing research regarding the association of the CNR1 gene with obesity and dyslipidemia is critical for the understanding of how the endocannabinoid system regulates overall energy expenditure in the human body. We believe further studies with bigger sample sizes are needed in order to confirm our findings.
